# Supplementary material for: A rotary mechanism for allostery in bacterial hybrid malic enzymes
Source: Nat Commun. 2021 Feb 23;12:1228. doi: 10.1038/s41467-021-21528-2 (PMC7902834; doi:10.1038/s41467-021-21528-2)
Supplement: Supplementary file 1 — Supplementary Information [file 41467_2021_21528_MOESM1_ESM.pdf]

Supplementary Information:

A Rotary Mechanism for Allostery in Bacterial Hybrid Malic Enzymes

Christopher John Harding, Ian Thomas Cadby, Patrick Joseph Moynihan, Andrew Lee Lovering*
Department of Biosciences, University of Birmingham, Birmingham, UK.

Contents:

Supplementary Figures
Supplementary Figure 1. Size Exclusion Chromatography Data
Supplementary Figure 2. Hybrid Malic Enzyme Sequence Alignment
Supplementary Figure 3. Comparison to eukaryotic malic enzymes and active PTA enzymes
Supplementary Figure 4. Electron Density Maps
Supplementary Figure 5. Inhibitor Profiling and Kinetic plots
Supplementary Figure 6. HPLC data
Supplementary Figure 7. Detail of Acetyl-CoA acetate group binding environment in the MaeB PTA domain.
Supplementary Figure 8. Design of MaeB$_{\Delta Hook}$ and its environment in full-length MaeB structures

Supplementary Table S1. Crystallographic Data Statistics
Supplementary Table S2. Kinetics Data
Supplementary Table S3. Primer sequences for molecular biology.
Supplementary Table S4. Crystallisation Conditions

Legend for Supplementary Movie S1

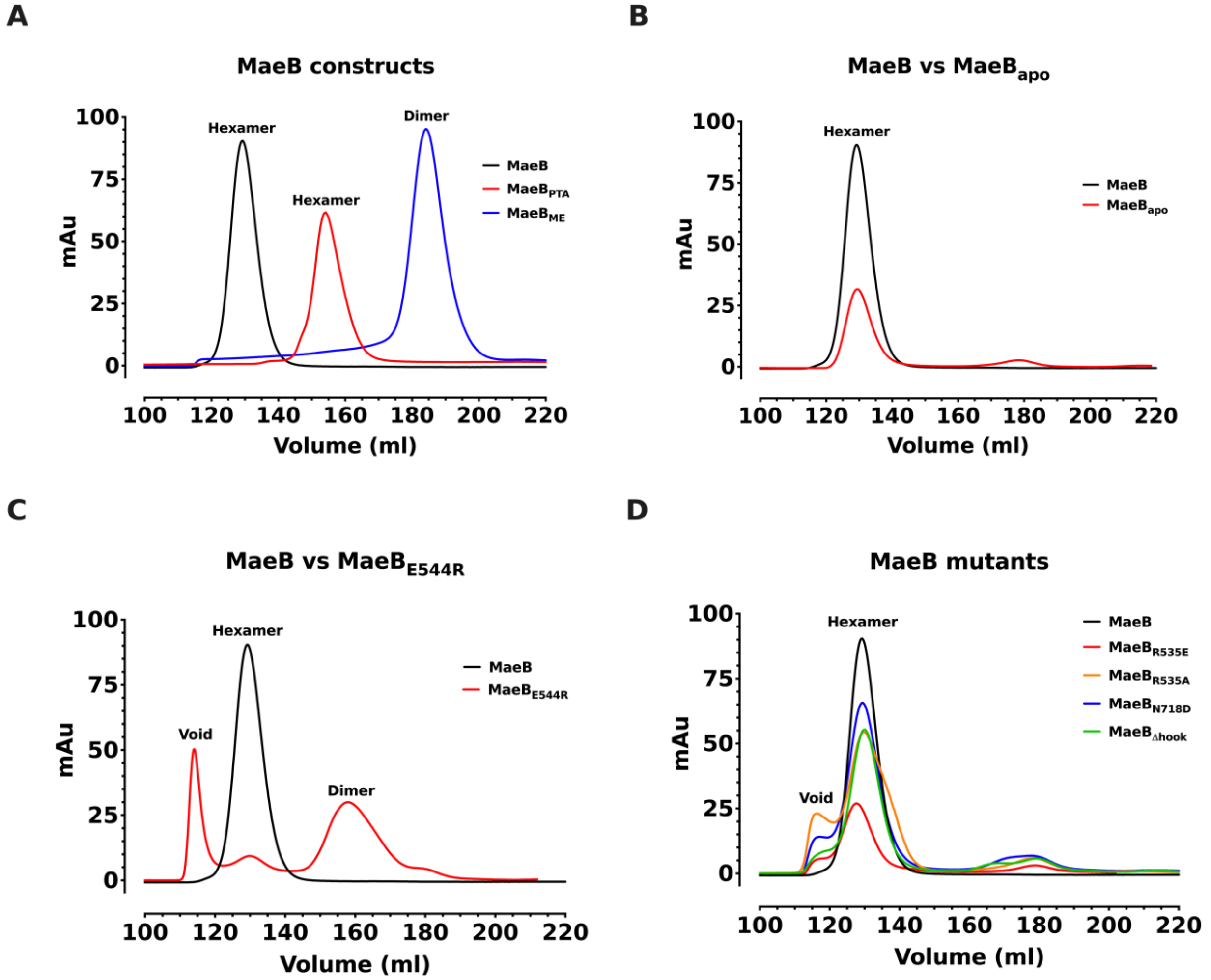

Supplementary Figure 1. Size Exclusion Chromatography Data

(A) Size exclusion chromatograms showing MaeB full-length & $MaeB_{PTA}$ elute as hexameric species and $MaeB_{ME}$ elutes as a dimeric species. (B) $MaeB_{apo}$ (washed with mild denaturing solution to remove acetyl-CoA) has a similar elution profile to native $MaeB_{bound}$. (C) MaeB-E544R mutant impedes hexamerisation and elutes as a dimeric species. (D) Comparison of size exclusion chromatograms for MaeB mutants that all elute as hexameric species. A peak in the void region is suggestive of the protein instability/aggregation.

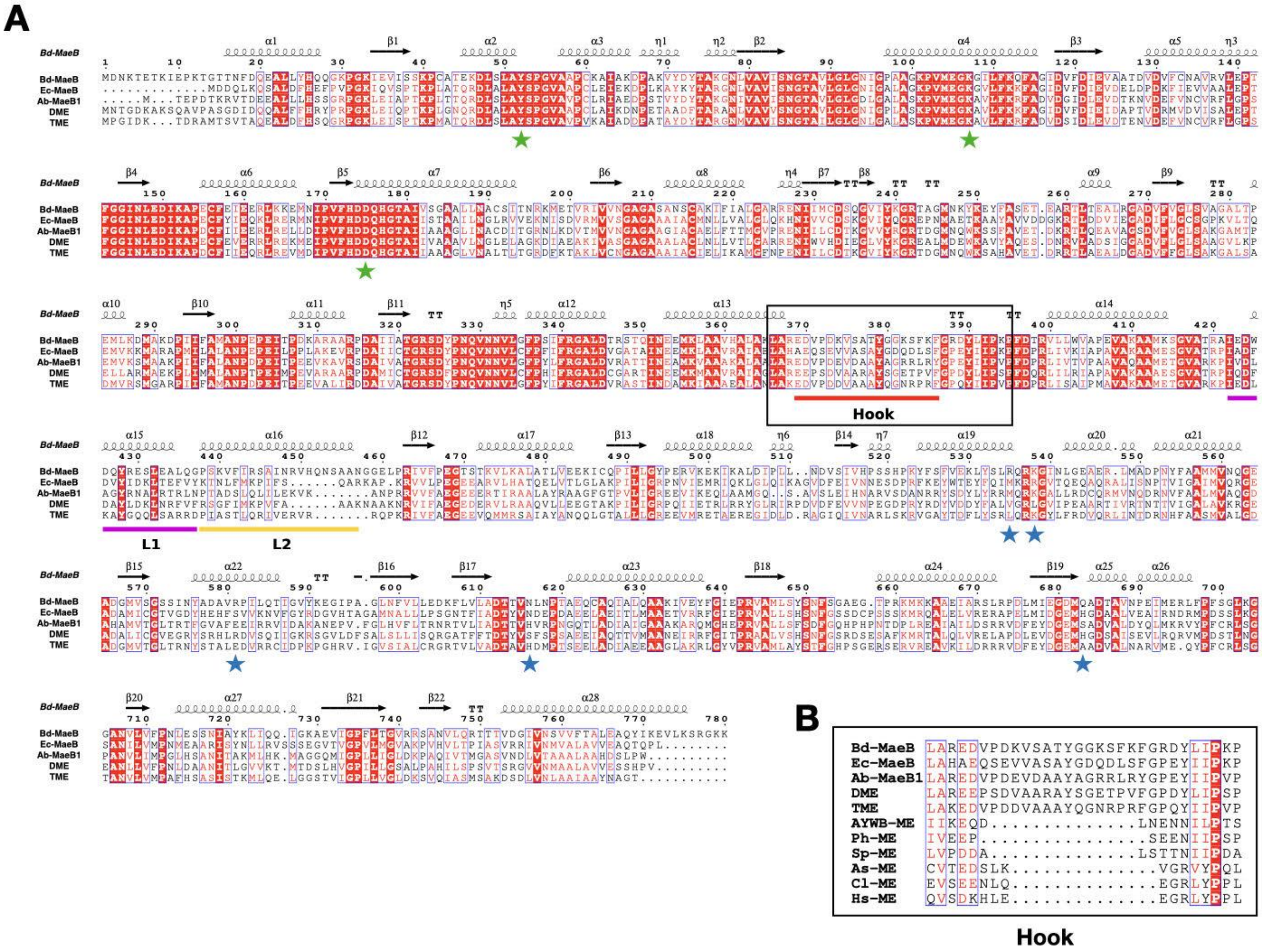

Supplementary Figure 2. Hybrid Malic Enzyme Sequence Alignment

(A) Sequence alignment of selected hybrid MEs. Green stars highlight catalytic residues and blue stars indicate the residues important for acetyl-CoA binding in *Bdellovibrio bacteriovorus* MaeB. The hybrid ME specific hook region (B, inset) is indicated by the red bar. L1 and L2 linker regions are shown by the pink and yellow bar, respectively.

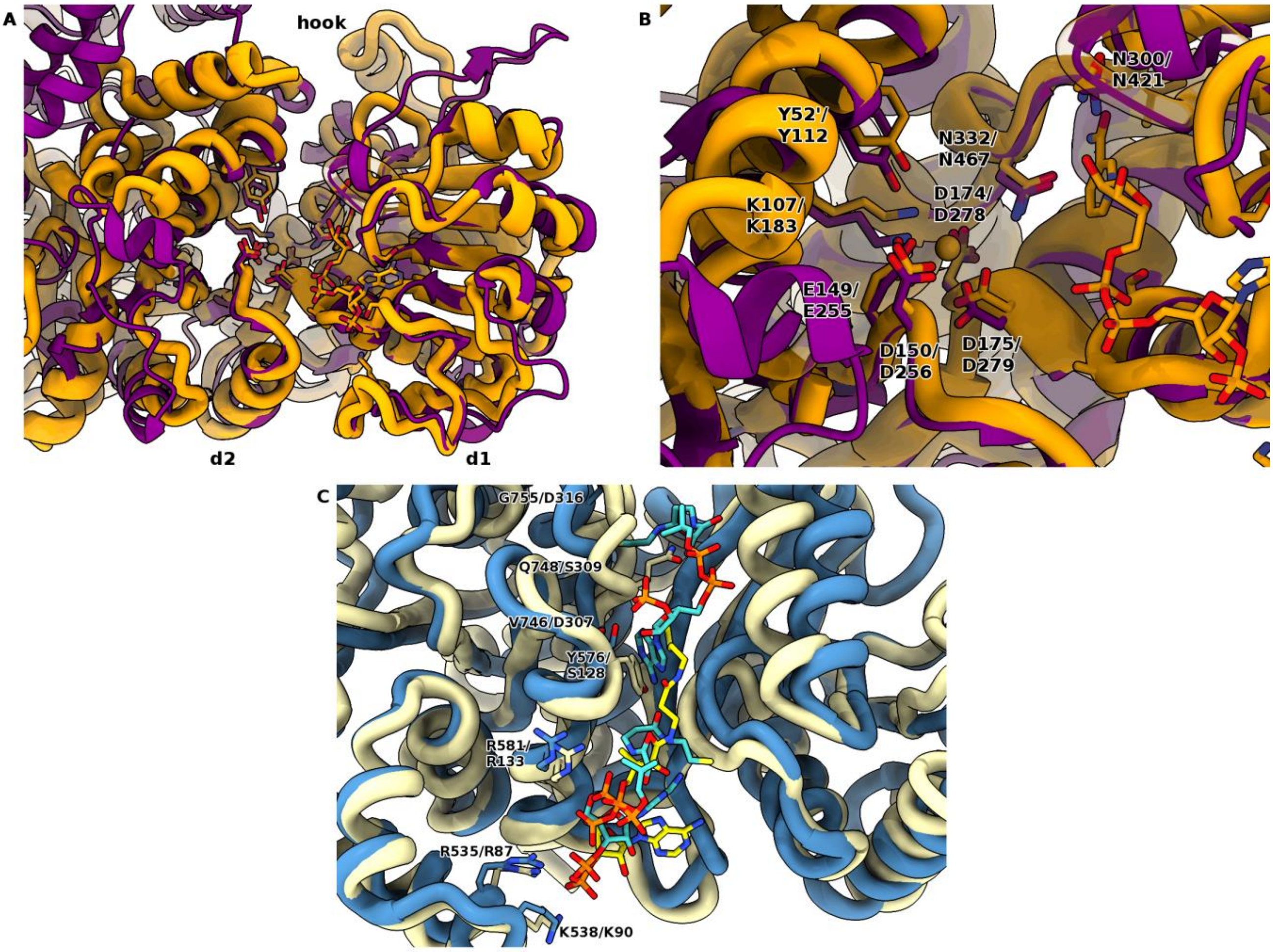

Supplementary Figure 3. Comparison to eukaryotic malic enzymes and active PTA enzymes (A) Superimposition of MaeB (orange) and eukaryotic malic enzyme (human mitochondrial NADP-specific enzyme, PDB: 1QR6, purple [1]). NADP and selected residues shown in stick form. MaeB d1 and d2 match well with the domains of eukaryotic enzymes, replacing the regulatory region at the upper face of the enzyme with the hook subdomain. (B) Structural equivalence of active site residues between MaeB and 1QR6, MaeB numbering is followed by 1QR6 equivalent, primed residues are contributed by the opposing monomer. (C) Superimposition of MaeB$_{PTA}$ domain bound state (beige, acetyl-CoA yellow) and a representative PTA enzyme (*Methanosarcina thermophila* PTA, PDB: 2AF3 [2], blue, two molecules of CoA cyan). Ligand and selected residues shown in stick form. The bound acetyl-CoA of MaeB occupies an equivalent space to the two molecules of CoA observed in the PTA enzyme complex, resulting in a different usage of conserved residues (MaeB numbering first, 2AF3 second). This results in a relatively greater closure of d1:d2 domains in MaeB – compare the equivalent loops and helices flanking the substrate binding cleft which runs top to bottom.

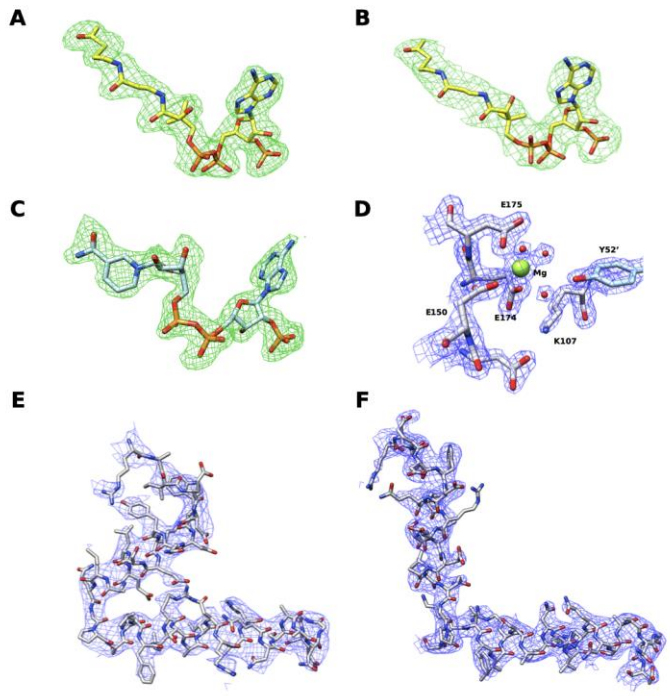

Supplementary Figure 4. Electron Density Maps

(A). Fo-Fc omit map for acetyl-CoA in the structure of $MaeB_{PTA}$. (B) Omit map for acetyl-CoA in the structure of full-length $MaeB_{bound}$. (C) Omit map for $NADP^+$ in the structure of $MaeB_{ME}$. (D) $2F_o$-F_c refined map density for the active site of MaeB. Maps A-D are inclusive of a 2 Å radius around the selected feature and contoured at 2 sigma. (E) The linker helix region of $MaeB_{apo}$. (F) The linker helix region of $MaeB_{bound}$. Panels E and F are from a $2F_o$-F_c refined map, set with a 2.5 Å radius and contoured at 1 sigma.

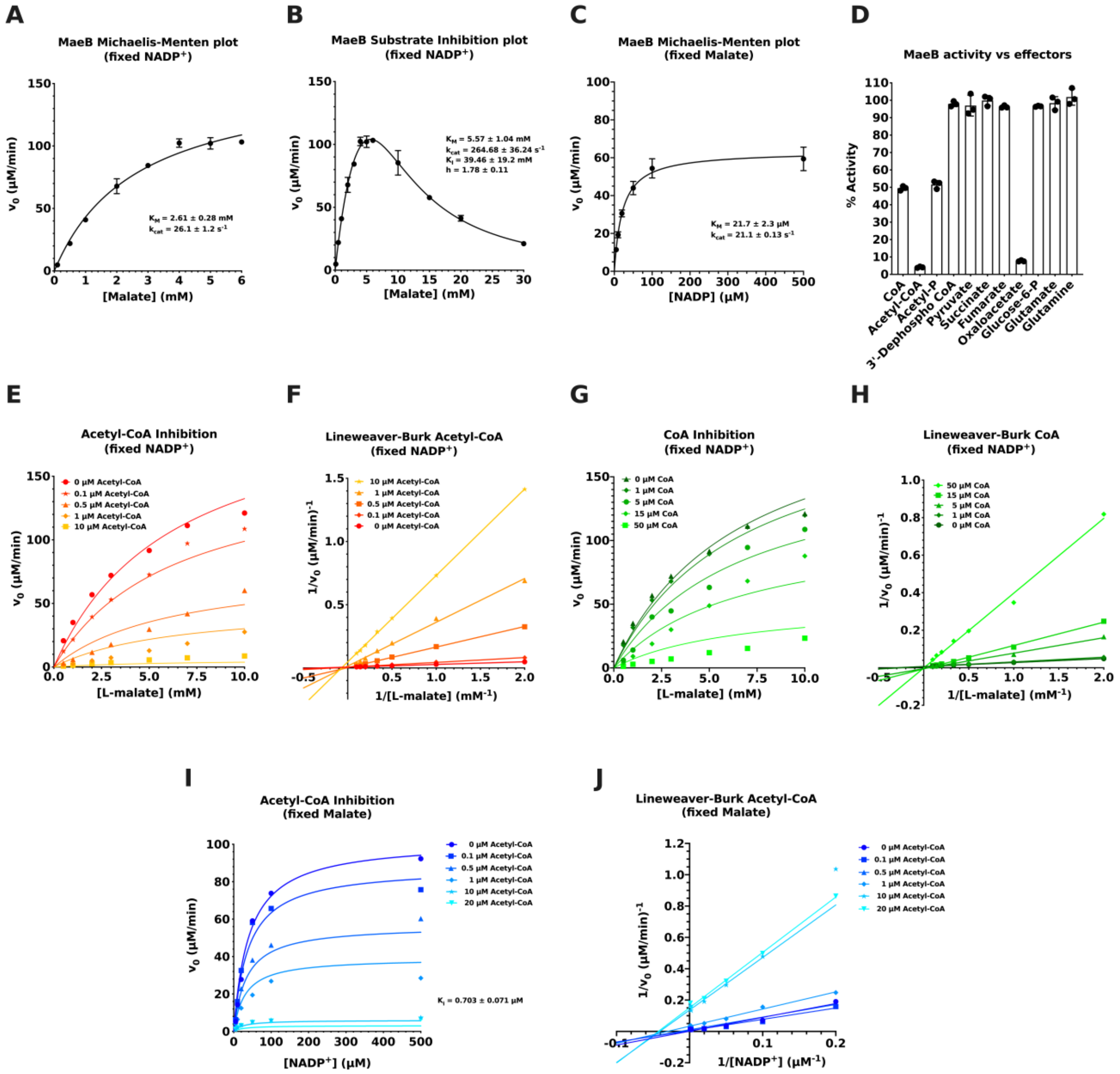

Supplementary Figure 5. Inhibitor Profiling and Kinetic plots

Data points represent the mean of three measurements and error bars represent the standard deviation. (A) Michaelis-Menten plot (fixed $NADP^+$ concentration) for MaeB full-length. (B) Michaelis-Menten plot (fixed L-malate concentration) for MaeB full length. (C) MaeB full-length – substrate inhibition is observed in the presence of high concentrations of L-malate. (D) The % activity of MaeB in the presence of a number of known malic enzyme effectors in comparison to standard reaction conditions. (E) Michaelis-Menten plots for MaeB in the presence of different acetyl-CoA concentrations (at fixed $NADP^+$ concentration). (F) Lineweaver-Burk plot for acetyl-CoA. (G) Michaelis-Menten plots for MaeB in the presence of different CoA concentrations (at fixed $NADP^+$ concentration). (H) Lineweaver-Burk plot for CoA. (I) Michaelis-Menten plots for MaeB in the presence of different acetyl-CoA concentrations (at fixed L-malate concentration). (J) Lineweaver-Burk plot for acetyl-CoA. Michaelis-Menten plots fit globally to a non-competitive inhibition model.

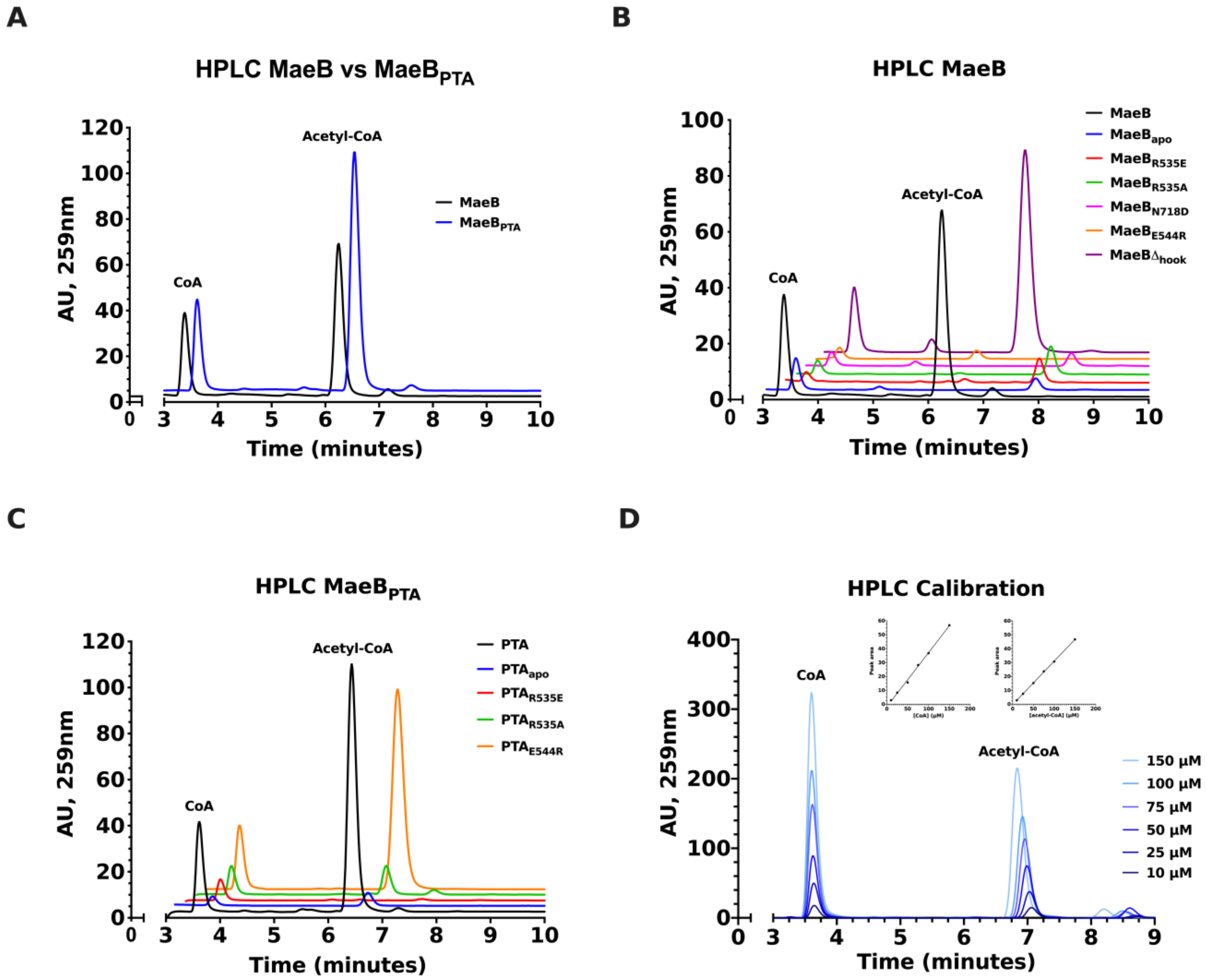

Supplementary Figure 6. HPLC data

(A) HPLC traces for wt MaeB full-length in comparison to wt MaeB$_{PTA}$ (100 µM samples injected). Both MaeB and MaeB$_{PTA}$ copurify predominantly with acetyl-CoA (~41 µM and ~66 µM, respectively), although both copurify with a smaller, yet similar amount of CoA (~18 µM). This finding indicates MaeB and MaeB$_{PTA}$ have, ~60% and ~85%, respectively, of the allosteric binding sites are occupied with ligand. (B) Comparison of full-length mutants/variants. MaeB$_{apo}$ is almost completely void of co-purified ligand (low levels of CoA and acetyl-CoA not detected), similarly to R535E and N718D mutants that prevent ligand binding. MaeB$_{\Delta hook}$ copurifies with comparatively similar amounts ligand to MaeB full-length. (C) Comparison of MaeB$_{PTA}$ mutants demonstrates R535 mutants and washed PTA$_{Apo}$, almost completely lack co-purified ligand. The HPLC traces are offset by 0.2 min and 2.5 AU, for clear illustration of the results. (D) CoA and acetyl-CoA were analysed by HPLC separately to ascertain their retention time. The HPLC assay was then calibrated by injecting known concentrations of a CoA/acetyl-CoA mixture. The area of each peak was then plotted against the concentration of CoA or acetyl-CoA and simple linear regression used to fit a best fit line to the datapoints. HPLC-UV was used to monitor the change in absorbance at λ_{max} for CoA (259 nm).

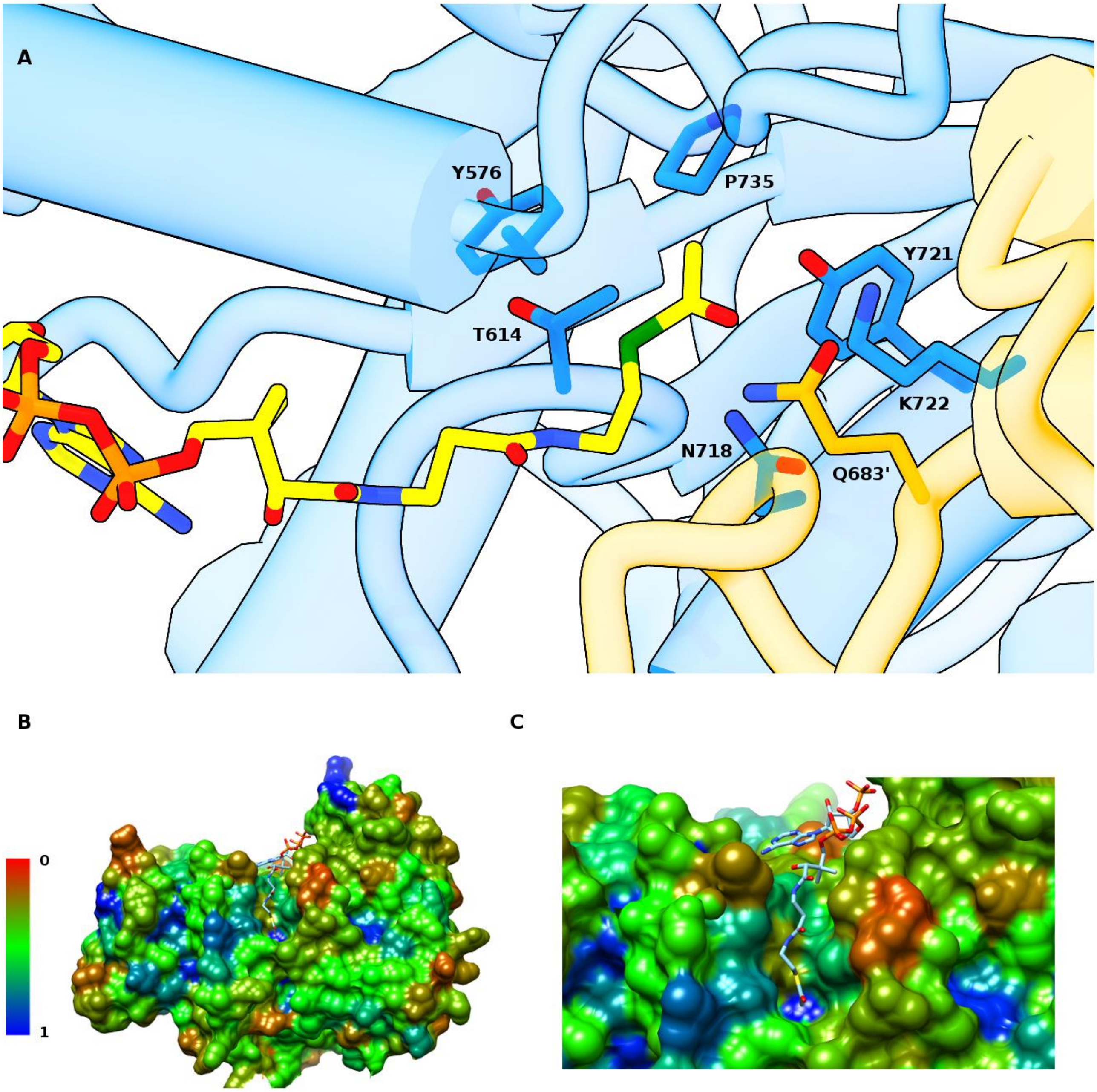

Supplementary Figure 7. Detail of Acetyl-CoA acetate group binding environment in the MaeB PTA domain.

(A) Acetyl-CoA is shown in stick form, C atoms yellow, between two PTA monomers (first coloured blue, select residues labelled non-prime; second coloured orange, residue labelled with prime). (B) Sequence conservation analysis of PTA domain, coloured from low (0, red) to high (1, blue) relative conservation; figure generated by CONSURF[3] analysis using ~5600 homologous sequences identified by HMMER[4], converted to an alignment of 150 representative members. (C) Zoomed-in version of (B) centred upon Acetyl-CoA pocket (ligand in stick form, C atoms coloured light blue).

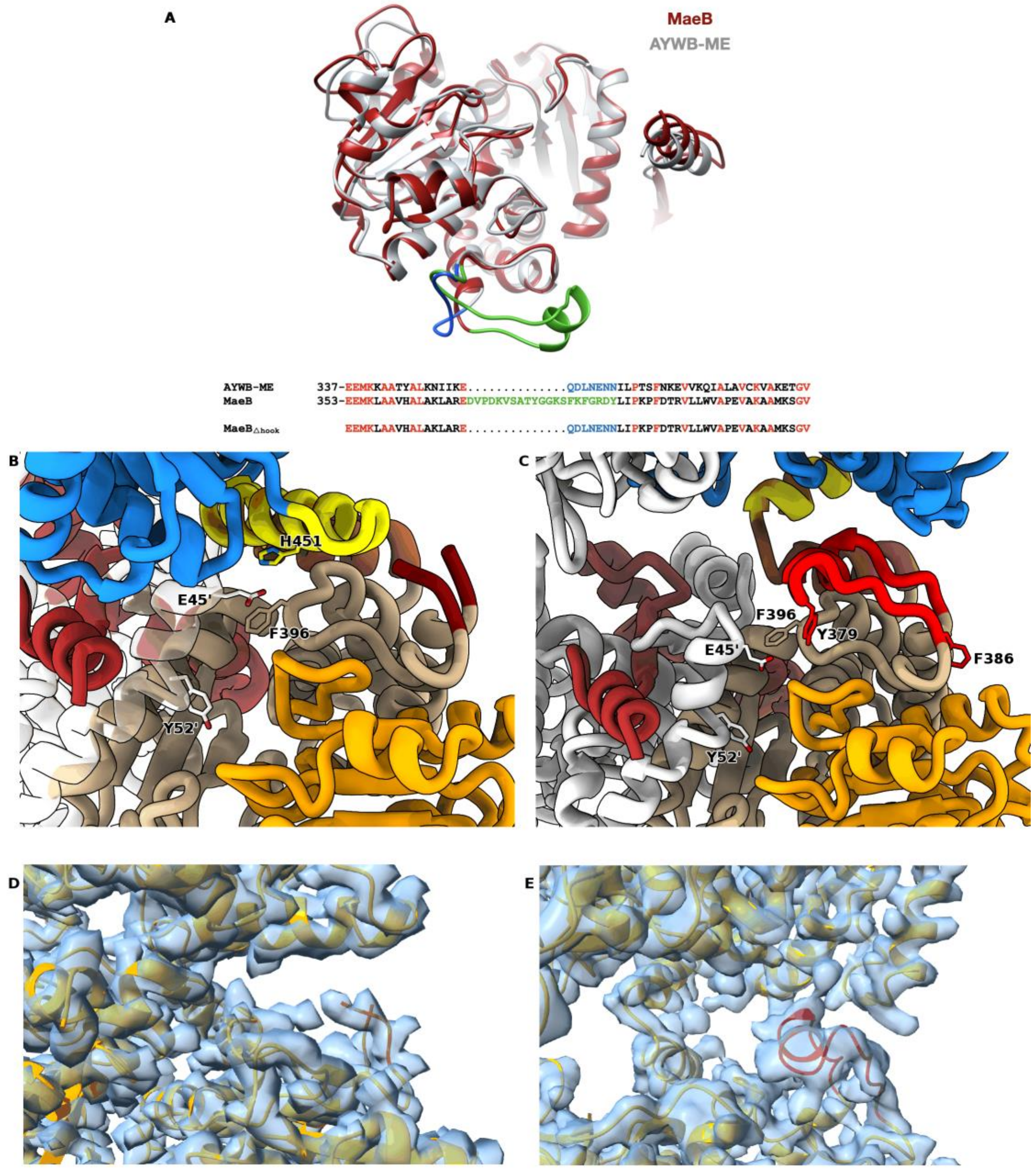

Supplementary Figure 8. Design of MaeB$_{\Delta\mathrm{Hook}}$ and its environment in full-length MaeB structures

(A) The chimera MaeB$_{\Delta\mathrm{Hook}}$ mutant was designed by superimposing the minimal ME, AYWB (PDB: 5CEE)[5], onto the structure of MaeB$_{\mathrm{ME}}$. The corresponding loop from AYWB (coloured blue) was swapped for the hook from MaeB (coloured green). (B) Acetyl-CoA bound form of MaeB, demonstrating that the active site helix contributed by the opposing monomer (primed residues, including catalytic Y52), makes contacts via E45 to H451 of the linker region. The hook subdomain (red) is partly disordered. (C) Apo form of MaeB, demonstrating that the hook folds as per the isolated ME domain structure, placing Y379 near E45. (D) and (E) Electron-density $2F_o$-F_c maps (final refinement, contoured at 1 sigma) showing difference in density for the hook subdomain region (red) between bound form (D) and apo-form (E).

Table S1. Crystallographic Data Statistics

*Numbers in parentheses refer to the outermost shell

	MaeB		$MaeB_{ME}$		$MaeB_{PTA}$					
	Native (Acetyl CoA)	Native (Apo)	Native (Apo)	Native ($NADP^+$)	Native (Acetyl CoA)	Native (Apo)	PTA_{R535A}	PTA_{R535E}	PTA_{N718D}	PTA_{E544R}
**Accession Code**	6ZNG	6ZNJ	6ZN4	6ZN7	6ZNT	6ZN9	6ZNR	6ZNE	6ZNK	6ZNU
**DataCollection**										
Spacegroup	C 2	C 2 2 2_1	P 2_1	P 2_1	P 2_1	P 2_1 2_1 2_1	C 2	C 2	P 2_1 2_1 2_1	C 2
**Unit Cell Dimensions**										
Cell Dimensions a, b, c (Å)	250, 145, 171	81.7, 273.6, 308.2	48.1, 92.7, 97.1	47.8, 92.2, 95.8	111.2, 60.6, 167.8	139.2, 151.3, 285.3	129.6, 183.7, 119.9	129.4, 183.4, 120.2	139.6, 150.8, 282.1	129.1, 182.9, 119.7
?, ?, ? (°)	90, 101, 90	90, 90, 90	90, 91.1, 90	90, 91.3, 90	90, 91.1, 90	90, 90, 90	90, 117, 90	90, 117, 90	90, 90, 90	90, 118, 90
Resolution (Å)	124.80- 2.72	154.12, 3.70	43.41- 1.67	95.78- 1.67	91.86- 1.96	142.66- 2.72	64.50- 2.35	107.10- 2.39	102.46- 3.04	69.22- 2.33
Total Reflections	1086009 (56926)	473316 (24400)	656294 (32403)	626225 (29211)	1083798 (55212)	2082343 (102453)	705212 (37173)	424649 (17692)	1456363 (95129)	698066 (266312)
Unique Reflections	160818 (8003)	36296 (1825)	96511 (4697)	96099 (4736)	160802 (7925)	162146 (7836)	103459 (5235)	96539 (4863)	115105 (8421)	104231 (5084)
Rmerge(I)	0.1 (2.788)	0.977 (7.796)	0.0851 (1.6100)	0.1191 (0.7974)	0.0589 (1.4047)	0.1294 (0.8860)	0.0867 (1.8559)	0.103 (1.975)	0.148 (1.946)	0.0872 (1.2654)
Rpim	0.042 (1.125)	0.280 (2.191)	0.036 (0.634)	0.050 (0.350)	0.025 (0.571)	0.038 (0.254)	0.036 (0.744)	0.055 (1.201)	0.063 (0.887)	0.036 (0.600)
I/sigI	11.0 (0.6)	4.6 (0.5)	10.81 (1.22)	6.87 (1.32)	15.70 (1.14)	11.40 (1.47)	10.69 (1.16)	8.5 (0.5)	10.5 (1.2)	12.03 (1.14)
CC(1/2)	0.999 (0.317)	0.963 (0.481)	0.9977 (0.4634)	0.9962 (0.4718)	0.999 (0.637)	0.998 (0.600)	0.999 (0.573)	0.998 (0.697)	0.998 (0.632)	0.999 (0.590)
Completeness (%)	99.9 (100)	97.1 (100)	97.80 (96.54)	99.90 (98.14)	99.92 (99.92)	99.95 (97.82)	99.90 (100.00)	98.4 (99.7)	100 (100)	99.72 (97.98)
Multiplicity	6.8 (7.1)	13.0 (13.4)	6.81 (6.98)	6.52 (6.17)	6.74 (6.97)	12.84 (13.07)	6.81 (7.10)	4.4 (3.6)	12.7 (11.3)	6.70 (5.24)
**Refinement**										
R-work (%)	19.22	20.70	0.1702	0.1799	0.1765	0.2520	0.2158	0.2153	21.34	0.2069
R-free (%)	22.77	27.66	0.2039	0.2159	0.2095	0.2995	0.2368	0.2602	25.45	0.2265
Average B Factor (Å)	108	103.20	37.00	34.50	57.57	85.78	60.40	75.50	94.70	74.90
No. of Atoms	34603	17400	6808	6862	16457	31187	15577	15628	31316	15863
Protein	34297	17400	6126	6149	15208	31187	15577	15581	31196	15551
Ligand / Ion	306	0	2	98	352	0	0	0	120	312
Solvent	0	0	680	615	591	0	0	47	0	0
Molecules per ASU	6	3	2	2	6	12	6	6	12	6
RMS (bonds) (Å)	0.0108	0.0137	0.0077	0.0108	0.009	0.012	0.0103	0.0100	0.0111	0.0097
RMS (angles) (°)	1.437	1.657	1.113	1.257	1.178	1.590	1.498	1.415	1.441	1.448
**Ramachandran Statistics**										
Favoured (%)	97.43	96.41	96.80	96.81	98.11	98.25	96.48	98.12	97.52	97.01
Allowed (%)	2.5	3.55	3.20	3.19	1.59	1.51	3.52	1.64	2.21	2.74
Outliers (%)	0.07	0.04	0.00	0	0.3	0.25	0	0.25	0.27	0.25

Table S2. Kinetics Data

Kinetic values were calculated from averages from triplicates measurements and fit using non-linear regression to the Michaelis-Menten equation. Reported with standard error values.

	Malate oxidative decarboxylation (L-Malate)			Malate oxidative decarboxylation ($NADP^+$)			Pyruvate reduction carboxylation (Pyruvate)		
	K_M (mM)	k_{cat} (s^{-1})	k_{cat}/K_M ($s^{-1}mM^{-1}$)	K_M (µM)	k_{cat} (s^{-1})	k_{cat}/K_M (s^{-1} µM^{-1})	K_M (mM)	k_{cat} (s^{-1})	k_{cat}/K_M ($s^{-1}mM^{-1}$)
**MaeB**	2.61 ± 0.28	26.1 ± 1.2	10.0	21.7 ± 2.3	21.1 ± 0.13	0.97	6.75 ± 0.70	0.21 ± 0.11	0.03
**$MaeB_{apo}$**	3.02 ± 0.31	35.2 ± 1.4	11.7	43.1 ± 5.2	33.5 ± 1.3	0.78			
**$MaeB_{R535E}$**	3.17 ± 0.15	19.4 ± 0.36	6.12	41.1 ± 4.0	16.8 ± 0.54	0.41			
**$MaeB_{R535A}$**	3.48 ± 0.23	20.3 ± 0.53	5.83						
**$MaeB_{N718D}$**	3.35 ± 0.33	41.8 ± 1.6	12.5						
**$MaeB_{E544R}$**	12.6 ± 2.0	18.9 ± 1.9	1.50						
**$MaeB_{ME}$**	4.15 ± 0.50	3.28 ± 0.17	0.79						
**$MaeB_{\Delta hook}$**	12.5 ± 1.0	1.62 ± 0.09	0.13						

Table S3. Primer sequences for molecular biology.

Construct	Primer Sequences
Bd1833	Fwd:<br>5’GCAGCGGCCTGGTGCCGCGCGGCAGCCATAtggacaacaaaactgaaactaaaattgaac-3’<br>Rev:<br>5’- ctcagtggtggtggtggtggtgctcgagttactttttgccacggcttttcaG-3’
Bd1833-PTA	Fwd:<br>5’- GCAGCGGCCTGGTGCCGCGCGGCAGCCATtccaaagtcttcatccgctctg-3’<br>Rev:<br>5’- ctcagtggtggtggtggtggtgctcgagttactttttgccacggcttttcaG-3’
Bd1833-ME	Fwd:<br>5’-<br>GCAGCGGCCTGGTGCCGCGCGGCAGCCATAtggacaacaaaactgaaactaaaattgaac-3’<br>Rev:<br>5’- ctcagtggtggtggtggtggtgctcgagctaagattcgcggtactgatccc-3’
Bd1833 R535A	Fwd: 5’- GTATTCTTTGgcCCAGCGTAAAGG-3’<br>Rev: 5’- AGCTTTTCAACGAAGCTG-3’
Bd1833 R535E	Fwd: 5’- GTATTCTTTGgagCAGCGTAAAGGC-3’<br>Rev: 5’- AGCTTTTCAACGAAGCTG-3’
Bd1833 E544R	Fwd: 5’- CAATCTGGGCagAGCCGAGCGC-3’<br>Rev: 5’- ATGCCTTTACGCTGGCGC-3’
Bd1833 N718D	Fwd: 5’- GGAATCCTCCgACATCGCCTA-3’<br>Rev: 5’- AGATTCGGGAAGACCAGC-3’
Bd1833 Δhook	Fwd: 5’- cttaacgagaacaacCTGATCCCGAAACCCTTC-3’<br>Rev: 5’- gtcttgctctttgatCAGTTTTGCCAGTGCATG-3’

Table S4. Crystallisation Conditions

		**Crystallisation Condition**
**MaeB**	**Native (acetyl CoA)**	0.2 M sodium citrate tribasic monohydrate, 0.1 M BisTris Propane pH 8.5, 20 % w/v PEG 3350
	**Native (Apo)**	0.1 M Tris pH 8.0, 20% v/v MPD
**MaeB$_{ME}$**	**Native (Apo)**	0.1 M Bicine pH 9.3, 25 % v/v PEG smear medium*
	**Native (NADP+)**	0.1 M Bicine pH 9.3, 25 % v/v PEG smear medium*
	**Native (Acetyl CoA)**	0.2 M TMAO, 0.1 M Tris pH 8.5, 20 % w/v PEG 2000 MME
	**Native (Apo)**	0.1 M magnesium acetate, 0.1 M sodium acetate pH 4.5, 8 % w/v PEG 8000
**MaeB$_{PTA}$**	**PTA$_{R535A}$**	0.2 M magnesium chloride hexahydrate, 10 % v/v ethylene glycol, 0.1 M HEPES pH 7.5,
	**PTA$_{R535E}$**	0.1 M magnesium chloride, 0.1 M HEPES pH 7.5, 10 % w/v PEG400, 15 % v/v PEG Smear Medium*, 5 % v/v 2-propanol
	**PTA$_{E544R}$**	0.2 M magnesium chloride hexahydrate, 10% v/v ethylene glycol, 0.1 M HEPES pH 7.5, 15 % v/v PEG Smear Medium, 5 % v/v 2-propanol
	**PTA$_{N718D}$**	0.1 M HEPES pH 7.0, 20 % w/v PEG 8000

*PEG smear medium (12.5% w/v PEG 2000, 12.5% w/v PEG 3350, 12.5% w/v PEG 4000, 12.5% w/v PEG 5000 MME)

Supplementary Movie S1

This movie demonstrates the large rotational conformational change that occurs between the apo- and inhibitor-bound forms of MaeB. The intermediate states were calculated using the morph conformations tool of Chimera[6], using our full-length structures as the end states.

Supplementary References

1 Xu, Y. W., Bhargava, G., Wu, H., Loeber, G. & Tong, L. Crystal structure of human mitochondrial NAD(P)(+)-dependent malic enzyme: a new class of oxidative decarboxylases. *Structure with Folding & Design* **7**, 877-889, doi:10.1016/s0969-2126(99)80115-4 (1999).

2 Lawrence, S. H., Luther, K. B., Schindelin, H. & Ferry, J. G. Structural and functional studies suggest a catalytic mechanism for the phosphotransacetylase from Methanosarcina thermophila. *J Bacteriol* **188**, 1143-1154, doi:10.1128/JB.188.3.1143-1154.2006 (2006).

3 Ashkenazy, H. *et al.* ConSurf 2016: an improved methodology to estimate and visualize evolutionary conservation in macromolecules. *Nucleic Acids Research* **44**, W344-W350, doi:10.1093/nar/gkw408 (2016).

4 Finn, R. D., Clements, J. & Eddy, S. R. HMMER web server: interactive sequence similarity searching. *Nucleic Acids Research* **39**, W29-W37, doi:10.1093/nar/gkr367 (2011).

5 Alvarez, C. E. *et al.* The crystal structure of the malic enzyme from Candidatus Phytoplasma reveals the minimal structural determinants for a malic enzyme. *Acta Crystallogr D Struct Biol* **74**, 332-340, doi:10.1107/S2059798318002759 (2018).

6 Pettersen, E. F. *et al.* UCSF chimera - A visualization system for exploratory research and analysis. *Journal of Computational Chemistry* **25**, 1605-1612, doi:10.1002/jcc.20084 (2004).
