# Supplementary material for: A rotary mechanism for allostery in bacterial hybrid malic enzymes
Source: Nat Commun. 2021 Feb 23;12:1228. doi: 10.1038/s41467-021-21528-2 (PMC7902834; doi:10.1038/s41467-021-21528-2)
Supplement: Supplementary file 4 — Description of Additional Supplementary Files [file 41467_2021_21528_MOESM4_ESM.pdf]

Description of Additional Supplementary Files

Title: Supplementary Movie 1

Description: This movie demonstrates the large rotational conformational change that occurs between the apo- and inhibitor-bound forms of MaeB. The intermediate states were calculated using the morph conformations tool of Chimera (Pettersen et al., 2004), using our full-length structures as the end states.
