# Supplementary material for: A rotary mechanism for allostery in bacterial hybrid malic enzymes
Source: Nat Commun. 2021 Feb 23;12:1228. doi: 10.1038/s41467-021-21528-2 (PMC7902834; doi:10.1038/s41467-021-21528-2)
Supplement: Supplementary file 5 — Reporting Summary [file 41467_2021_21528_MOESM5_ESM.pdf]

nature research

Corresponding author(s): Dr Andrew L. Lovering

Last updated by author(s): Jan 27, 2021

Reporting Summary

Nature Research wishes to improve the reproducibility of the work that we publish. This form provides structure for consistency and transparency in reporting. For further information on Nature Research policies, see our Editorial Policies and the Editorial Policy Checklist.

Statistics

For all statistical analyses, confirm that the following items are present in the figure legend, table legend, main text, or Methods section.

n/a	Confirmed	
☐	☒	The exact sample size (*n*) for each experimental group/condition, given as a discrete number and unit of measurement
☐	☒	A statement on whether measurements were taken from distinct samples or whether the same sample was measured repeatedly
☒	☐	The statistical test(s) used AND whether they are one- or two-sided<br>*Only common tests should be described solely by name; describe more complex techniques in the Methods section.*
☒	☐	A description of all covariates tested
☐	☒	A description of any assumptions or corrections, such as tests of normality and adjustment for multiple comparisons
☐	☒	A full description of the statistical parameters including central tendency (e.g. means) or other basic estimates (e.g. regression coefficient) AND variation (e.g. standard deviation) or associated estimates of uncertainty (e.g. confidence intervals)
☒	☐	For null hypothesis testing, the test statistic (e.g. *F*, *t*, *r*) with confidence intervals, effect sizes, degrees of freedom and *P* value noted<br>*Give P values as exact values whenever suitable.*
☒	☐	For Bayesian analysis, information on the choice of priors and Markov chain Monte Carlo settings
☒	☐	For hierarchical and complex designs, identification of the appropriate level for tests and full reporting of outcomes
☒	☐	Estimates of effect sizes (e.g. Cohen's *d*, Pearson's *r*), indicating how they were calculated

Our web collection on statistics for biologists contains articles on many of the points above.

Software and code

Policy information about availability of computer code

Data collection	Kinetics data was collected using CARY WinUV kinetics software<br>Size exclusion data collected using AKTA UNICORN 7.0 software<br>HPLC data was collected using Chromeleon™ Chromatography Data System (CDS) Software<br>Structural data was collect at Diamond Light source using the ISPyB server
Data analysis	Kinetics, SEC and HPLC data analyzed using Prism 8 software<br>Structural data was analysed using the CCP4i suit and Phenix

For manuscripts utilizing custom algorithms or software that are central to the research but not yet described in published literature, software must be made available to editors and reviewers. We strongly encourage code deposition in a community repository (e.g. GitHub). See the Nature Research guidelines for submitting code & software for further information.

Data

Policy information about availability of data

All manuscripts must include a data availability statement. This statement should provide the following information, where applicable:

- Accession codes, unique identifiers, or web links for publicly available datasets
- A list of figures that have associated raw data
- A description of any restrictions on data availability

Coordinates and structure factors have been deposited in the PDB under accession codes:
PDB: 6ZNJ [http://doi.org/10.2210/pdb6ZNJ/pdb] (full-length apo form),
PDB: 6ZNG [http://doi.org/10.2210/pdb6ZNG/pdb] (full-length acetyl-CoA bound form),
PDB: 6ZN4 [http://doi.org/10.2210/pdb6ZN4/pdb] (MaeBME apo form),

PDB: 6ZN7 [http://doi.org/10.2210/pdb6ZN7/pdb] (MaeBME NADP+ complex),
PDB: 6ZN9 [http://doi.org/10.2210/pdb6ZN9/pdb] (MaeBPTA apo form),
PDB: 6ZNT [http://doi.org/10.2210/pdb6ZNT/pdb] (MaeBPTA acetyl-CoA bound form),
PDB: 6ZNR [http://doi.org/10.2210/pdb6ZNR/pdb] (MaeBPTA R535A),
PDB: 6ZNE [http://doi.org/10.2210/pdb6ZNE/pdb] (MaeBPTA R535E),
PDB: 6ZNK [http://doi.org/10.2210/pdb6ZNK/pdb] (MaeBPTA N718D)
PDB: 6ZNU (MaeBPTA E544R).
Source data (raw data) has been provided for the following figures: Figure 2, Sup fig. 1, Sup fig. 5, Sup fig. 6, Table S2

Field-specific reporting

Please select the one below that is the best fit for your research. If you are not sure, read the appropriate sections before making your selection.

☒ Life sciences ☐ Behavioural & social sciences ☐ Ecological, evolutionary & environmental sciences

For a reference copy of the document with all sections, see nature.com/documents/nr-reporting-summary-flat.pdf

Life sciences study design

All studies must disclose on these points even when the disclosure is negative.

Sample size	N/A for the experiments performed
Data exclusions	No data was excluded from analysis
Replication	Kinetics data, each experiment was performed in at least triplicate, all attempts successful. HPLC data, collected in at least duplicates. Measurements carried out over multiple days, standards ran with each batch to monitor baseline drift due to new mobile phase formulations, replicates all successful. Size exclusion data, collected during purification process of each protein. Each protein purified on at least two independent occasions, which gave comparable SEC traces.
Randomization	N/A for the experiments performed
Blinding	N/A for the experiments performed

Reporting for specific materials, systems and methods

We require information from authors about some types of materials, experimental systems and methods used in many studies. Here, indicate whether each material, system or method listed is relevant to your study. If you are not sure if a list item applies to your research, read the appropriate section before selecting a response.

Materials & experimental systems

n/a	Involved in the study	
☒	☐	Antibodies
☒	☐	Eukaryotic cell lines
☒	☐	Palaeontology and archaeology
☒	☐	Animals and other organisms
☒	☐	Human research participants
☒	☐	Clinical data
☒	☐	Dual use research of concern

Methods

n/a	Involved in the study	
☒	☐	ChIP-seq
☒	☐	Flow cytometry
☒	☐	MRI-based neuroimaging
